# Analysis of Associations of Human BAFF Gene Polymorphisms with Autoimmune Thyroid Diseases

**DOI:** 10.1371/journal.pone.0154436

**Published:** 2016-05-02

**Authors:** Jiunn-Diann Lin, Shun-Fa Yang, Yuan-Hung Wang, Wen-Fang Fang, Ying-Chin Lin, Yuh-Feng Lin, Kam-Tsun Tang, Mei-Yi Wu, Chao-Wen Cheng

**Affiliations:** 1 Graduate Institute of Clinical Medicine, College of Medicine, Taipei Medical University, Taipei, Taiwan; 2 Division of Endocrinology, Department of Internal Medicine, Shuang-Ho Hospital, Taipei Medical University, New Taipei City, Taiwan; 3 Division of Endocrinology and Metabolism, Department of Internal Medicine, School of Medicine, College of Medicine, Taipei Medical University, Taipei, Taiwan; 4 Institute of Medicine, Chung Shan Medical University, Taichung, Taiwan; 5 Department of Medical Research, Chung Shan Medical University Hospital, Taichung, Taiwan; 6 Department of Medical Research, Shuang-Ho Hospital, Taipei Medical University, New Taipei City, Taiwan; 7 Department of Family Medicine, Shuang-Ho Hospital, Taipei Medical University, New Taipei City, Taiwan; 8 Division of Nephrology, Department of Internal Medicine, Shuang-Ho Hospital, Taipei Medical University, New Taipei City, Taiwan; 9 Division of Endocrinology and Metabolism, Department of Internal Medicine, Veterans General Hospital, Taipei, Taiwan; Hokkaido University, JAPAN

## Abstract

**Background:**

The B-lymphocyte-activating factor (BAFF) is associated with B-cell functions, and gene polymorphisms of the BAFF have been linked to autoimmune diseases (AIDs). In this study, we explored possible associations of two BAFF single-nucleotide polymorphisms (SNPs), rs1041569 and rs2893321, with autoimmune thyroid diseases (AITDs) in an ethnic Chinese population.

**Material and Methods:**

In total, 319 Graves’ disease (GD), 83 Hashimoto’s thyroiditis (HT) patients, and 369 healthy controls were enrolled. Polymerase chain reaction-restriction fragment length polymorphism and direct sequencing were used to genotype rs2893321 and rs1041569.

**Results:**

There was a significant difference in frequencies of the G allele and AG+GG genotype of rs2893321 between the GD and control groups (*p* = 0.013, odds ratio (OR) = 0.76, and *p* = 0.017, OR = 0.68, respectively) and between the AITD and control groups (*p* = 0.009, OR = 0.76, and, *p* = 0.014, OR = 0.69, respectively). The AA genotype of rs2893321 was associated with low titers of the thyroid-stimulating hormone receptor antibody (TSHRAb) (*p* = 0.015) in males but not in females. The AA genotype of rs2893321 was associated with the presence of two different types of thyroid autoantibody (TAb) (TSHRAb and Hashimoto’s autoantibody (anti-thyroglobulin or anti-microsomal antibody)) in females and with that of one type in males.

**Conclusions:**

rs2893321 may be a susceptible genetic variant for the development of GD and AITDs. Associations of rs2893321 with susceptibility to GD and AITDs and the correlation between rs2893321 and TAb exhibit a dimorphic pattern. Additional studies with larger sample sizes are required to confirm our findings.

## Introduction

B cells play a critical role in maintaining a normal adaptive immune response, through modulating T-cell functions, antibody formation, and inflammatory cytokine production [1]. B-Lymphocyte-activating factor (BAFF), a member of the tumor necrosis factor (TNF) family and which is regulated by type 1 interferon (IFN), is regarded as an essential factor for B-cell development and differentiation [2]. By binding to the BAFF receptor of B-cell membranes, the BAFF stimulates B cell maturation and proliferation and prolongs survival [3]. Evidence shows that a decline in the BAFF leads to a B-cell deficiency, while adding the BAFF to the circulation facilitates B-cell proliferation and increased serum antibody levels [4].

Evidence shows that BAFF dysregulation can contribute to immune disorders. In animal studies, BAFF overexpression in transgenic mice led to peripheral B-cell proliferation and lupus-like autoimmune features [5]. In humans, several studies showed that serum BAFF was increased in several autoimmune diseases (AIDs), including systemic lupus erythematous (SLE), rheumatoid arthritis (RA), primary biliary cirrhosis, and Sjögren's syndrome (SS) [6,7,8]. In addition, serum BAFF levels were reported to be related to circulating autoantibody concentrations [7,9]. At the same time, several genetic studies also documented that BAFF genetic variants were associated with the occurrence and phenotypes of AIDs [10,11,12,13,14,15,16].

Autoimmune thyroid disease (AITD), the most prevalent AID in the general population [17], comprises two major diverse types, Graves’ disease (GD) and Hashimoto’s thyroiditis (HT). GD is well known to mainly be associated with B-cell-predominant immunity [18]. In the meantime, although the occurrence of HT is mainly based on a T cell-driven pathogenesis, it is also involved in B-cell activation and autoantibody production [17]. In a mouse model of GD, inhibition of the BAFF with an anti-BAFF antibody reduced the thyroid-stimulating hormone antibody (TSHRAb) titer and thyroid function [19]. In human studies, Fabris et al. found that serum BAFF concentrations were high in both GD and HT patients compared to healthy subjects [20]. Vannucchi et al. observed an elevated serum BAFF level in patients with GD and also found a decline in the BAFF level after treatment with steroids [21]. This evidence suggested a close relationship between the BAFF and AITD; however, so far, associations of genetic variants of BAFF with the occurrence and clinical features of GD and HT in humans have not been reported despite the predominant genetic background of the pathogenesis.

In this study, we investigated possible associations of two single-nucleotide polymorphisms (SNPs) of the BAFF with AITD in an ethnic Chinese population. In addition, differences in clinical characteristics between these genetic variants, including thyroid function and basal thyroid autoantibody (TAb) titers, in GD and HT patients were also evaluated.

## Materials and Methods

### Subjects

Blood samples of 319 patients with GD and 83 patients with HT who were older than 20 years were collected by the Division of Endocrinology, Internal Department, Shuang-Ho Hospital (New Taipei City, Taiwan) from January 2013 to September 2014. In total, 369 blood samples of subjects older than 20 years without AITD or other AIDs were obtained by the Health Screening Center of Shuang-Ho Hospital from May to August 2014. AITD patients and healthy controls were excluded if they had any of the following criteria: younger than 20 years, pregnant, alcoholism, or a history of drug intoxication. The study protocol was approved by the Taipei Medical University-Joint Institutional Review Board, and all subjects provided written informed consent prior to participation.

GD was determined by one of the following criteria: (1) the existence of low thyroid-stimulating hormone (TSH), and either normal or high free thyroxine (FT4), and the existence of the TSH receptor antibody (TSHRAb); (2) the presence of thyrotoxicosis without TSHRAb but increased or normal diffuse thyroid uptake of I^131^; or (3) a proven diagnosis by another hospital according to medical records. HT was determined as the existence of Hashimoto’s autoantibody (either an anti-microsomal antibody (AmiA) or anti-thyroglobulin antibody (ATA) or both) in combination with hypoechogenic thyroid parenchyma found by a thyroid sonogram.

### Genotyping

Genomic DNA was obtained from 3 ml of whole-blood samples using a DNA blood extraction kit (Geneaid). Genetic variants of rs1041569 and rs2893321 were genotyped with a polymerase chain reaction-restriction fragment length polymorphism (PCR-RFLP) method. PCR amplification was performed using 1 μl DNA, 1 μl primer, 10 μl Tag PCR MasterMix (Genomics Biosci & Tech, Taiwan) and 7 μl H_2_O. PCR conditions of the two SNPs were as follows: denaturation at 94°C for 30 s, annealing at 55°C for 30 s, and extension at 72°C for 30 s for 35 cycles, with a final 7-min extension at 72°C. PCR products were incubated with restriction enzymes (New England Biolabs) at 37°C for 4 h. After incubation, PCR products were detected by electrophoresis with a 3% agarose gel. Primers and restriction enzymes for the two SNPs are shown in Table A in S1 File. About 10% of unrelated samples were randomly selected to repeat genotyping to exclude digestion errors, and no genotyping error was found. At the same time, several PCR products were directly sequenced for quality control (Figures A and B in S1 File).

### Laboratory analyses

Serum-free T4 and TSH were determined by an electrochemiluminescence immunoassay using commercial Roche Elecsys reagent kits (Roche Diagnostica). The normal range of free T4 is 0.93~1.7 ng/dl (with an intra-assay coefficient of variation (CV) of < 2.0%, and an inter-assay CV of < 4.8%), and that of TSH is 0.27~4.20 mU/L (with an intra-assay CV of < 3.0%, and an inter-assay CV of < 7.2%).

Serum AmiA and ATA concentrations were measured with an enzyme-linked immunosorbent assay (ELISA) using available commercial ImmunoCAP kits (Phadia, Taiwan). Titers of ≥ 280 IU/ml for ATA and ≥ 60 IU/ml for AmiA were defined as positive results. Serum TSHRAb concentrations were quantified by a radioimmunoassay method using a commercial TSH receptor autoantibody-coated tube kit (R.S.R., Ltd, United Kingdom). Data are expressed as the percentage of blocking of I^125^-labelled TSH binding to the TSH receptor coated onto a test tube [22]. A value of > 15% was defined as positive.

Serum BAFF levels were determined by a commercial ELISA kit (R&D Systems, Minneapolis, MN, USA) according the protocol of the manufacturer. Serum samples were diluted by 1:3 fold. Results are expressed as pg/ml.

### Statistical analysis

SPSS vers. 13.0 for Windows (SPSS, Chicago, IL, USA) was used for all statistical analyses. Quantitative values are shown as the mean ± standard deviation (SD). An independent *t*-test was used to compare differences of demographic data, FT4, TSH, and TSHRAb between two groups. A χ^2^ test was used to assess the difference in the GD, HT, or AITD groups with the control group. A one-way analysis of variance (ANOVA) was used to compare differences in clinical parameters among the GD, HT, and control groups. The Bonferroni test was used for post-hoc examinations. A test of Hardy-Weinberg equilibrium (HWE) was performed with the χ^2^ test. The χ^2^ test or Fisher’s exact test was also used to assess differences in categorical data between two groups. Patients with AITD were arbitrarily stratified into low and high AmiA groups according to the median value of the serum AmiA level. Associations of the susceptibility to either GD, HT, or AITD with clinical data, including SNPs, and demographic data were first evaluated by a univariate logistic regression. For instance, when AITD was defined as the dependent variable (0 for the control, 1 for AITD), the variables of interest, i.e., SNPs, age, gender, a family history (FH) of thyroid diseases, and smoking, were taken as independent variables. Significant discriminating factors were selected and further included in the multivariate logistic regression for analysis. All statistical tests were two-sided, and a *p* value of < 0.05 was considered significant.

## Results

### Demographic data of the GD, HT, AITD, and control groups

Demographic characteristics of the HT, GD, AITD, and control groups are shown in Table 1. Patients in the HT group were older than those in the control group, while there was a higher percentage of females in the HT than in the GD and control groups. Patients with GD had a higher prevalence of smoking than that in the control group. Patients in both the GD and HT groups had higher prevalence of FH than that in the control group. Patients in the AITD group were older and had higher percentages of females, a smoking habit, and FH than those in the control group.

**Table 1:** Demographic characteristics in the Graves’ disease (GD), Hashimoto’s thyroiditis (HT), and control groups.

	Control	GD	HT	AITD
Age (years)	43.3±11.5^c^^,^^d^	44.8±12.5	49.8±13.3^a^	45.8±12.9^a^
Gender (female %)	65.7^c^^,^^d^	70.1	90.4^a^	74.3^a^
Smoking (%)	14.9^b^^,^^d^	23.2^a^	17.3	22.0^a^
Family history of thyroid disease (%)	6.4^b^^,^^c^^,^^d^	29.6^a^	20.0^a^	27.6^a^

Age is expressed as the mean±standard deviation.

AITD, autoimmune thyroid disease.

^a^ *p* < 0.05 vs. the control group

^b^ *p* < 0.05 vs. GD

^c^ *p* < 0.05 vs. HT

^d^ *p* < 0.05 vs. AITD.

### SNP association analysis

In the current study, two SNPs were explored by PCR-RFLP, including one SNP in the 5’ regulatory region (rs1041569) [10] and another in intron 3 (rs2893321) [12] of the BAFF. Genotype frequencies of rs1041569 and rs2893321 were determined in all groups, and both of them were in HWE (data not shown). Differences in genotype and allelic frequencies of the two SNPs in the GD, HT, AITD, and control groups were compared, and results are respectively shown in Table B in S1 File and Table 2. There were significantly decreased frequencies of the G allele of rs2893321 in the GD group (*p* = 0.013, odds ratio (OR) = 0.76, 95% confidence interval (CI) = 0.61~0.94) and in the AITD group (*p* = 0.009, OR = 0.76, 95% CI = 0.62~0.94) compared to the control group. At the same time, there were also decreased percentages of the AG+GG genotype in the GD (*p* = 0.016, OR = 0.68, 95% CI = 0.50~0.93) and AITD (*p* = 0.014, OR = 0.69, 95% CI = 0.51~0.93) groups compared to the control group (Table 2). Interestingly, we observed that there were similar frequencies of the G allele and AG+GG genotype of rs2893321 in HT as in GD despite there being no statistical significance (*p* = 0.142, OR = 0.77, 95% CI = 0.55~1.10 and *p* = 0.192, OR = 0.72, 95% CI = 0.44~1.18, respectively), which suggests that rs2893321 might also be a susceptible genetic variant for the occurrence of HT.

**Table 2:** Genotype and allele frequencies of rs2893321 in the B-lymphocyte activating factor gene.

Polymorphism	Control *n* (%)	GD *n* (%)	HT *n* (%)	AITD *n* (%)	OR1 (95% CI)	OR2 (95% CI)	OR3 (95% CI)
rs2893321							
AA	111 (30.0)	123 (38.7)	31 (37.3)	154 (38.4)	1	1	1
AG	191 (51.6)	151 (47.5)	41 (49.4)	192 (47.9)	0.71 (0.51~1.00)*	0.77 (0.46~1.30)	0.76 (0.62~0.94)*
GG	68 (18.4)	44 (13.8)	11 (13.3)	55 (13.7)	0.58 (0.37~0.92)*	0.58 (0.57~1.23)	0.58 (0.38~0.90)*
AG+GG	259 (70.0)	195 (61.3)	52 (62.7)	247 (61.6)	0.68 (0.50~0.93)*	0.72 (0.44~1.18)	0.69 (0.51~0.93)*
Allele							
A	413 (55.8)	397 (62.4)	103 (62.0)	500 (62.3)	1	1	1
G	327 (44.2)	239 (37.6)	63 (38.0)	302 (37.7)	0.76 (0.61~0.94)*	0.77 (0.55~1.10)	0.76 (0.62~0.94)*
Female							
AA	74 (30.5)	92 (41.3)	26 (34.7)	118 (39.6)	1	1	1
AG	125 (51.4)	104 (46.6)	38 (50.7)	142 (47.7)	0.67 (0.45~1.00)*	0.85 (0.59~1.20)	0.71 (0.49~1.04)
GG	44 (18.1)	27 (12.1)	11 (14.7)	38 (12.8)	0.49 (0.28~0.87)*	0.71 (0.32~1.58)	0.54 (0.32~0.91)*
AG+GG	169 (69.5)	131 (58.7)	49 (65.4)	180 (60.5)	0.62 (0.43~0.91)*	0.83 (0.48~1.43)	0.67 (0.47~0.96)*
Allele							
A	273 (56.1)	288 (64.6)	90 (60.0)	378 (63.4)	1	1	1
G	213 (43.9)	158 (35.4)	60 (40.0)	218 (36.6)	0.70 (0.54~0.92)*	0.85 (0.58~1.24)	0.74 (0.58~0.94)*
Male							
AA	37 (29.1)	31 (32.6)	5 (62.5)	36 (35.0)	1	1	1
AG	66 (52.0)	47 (49.5)	3 (37.5)	50 (48.5)	0.85 (0.46~1.56)	0.34 (0.08~1.49)	0.78 (0.43~1.40)
GG	24 (18.9)	17 (17.9)	0 (0.0)	17 (16.5)	0.85 (0.39~1.85)	-	0.73 (0.34~1.58)
AG+GG	90 (70.9)	64 (67.4)	3 (37.5)	67 (65.0)	0.85 (0.48~1.51)	0.14 (0.01~2.63)	0.77 (0.44~1.34)
Allele							
A	140 (55.1)	109 (57.4)	13 (81.3)	122 (59.2)	1	1	1
G	114 (44.9)	81 (42.6)	3 (18.7)	84 (40.8)	0.91 (0.63~1.33)	0.28(0.1~1.02)	0.85 (0.58~1.23)

GD, Graves’ disease; HT, Hashimoto’s thyroiditis; AITD, autoimmune thyroid disease (Graves’ disease + Hashimoto’s thyroiditis); control, control group.

OR1, odds ratio 1, GD vs. the control.

OR2, odds ratio 2, HT vs. the control.

OR3, odds ratio 3, AITD vs. the control.

CI, confidence interval.

* *p* < 0.05.

There were decreased frequencies of the G allele of rs2893321 in the GD (*p* = 0.009, OR = 0.70, 95% CI = (0.54~0.92) and AITD groups (*p* = 0.015, OR = 0.74, 95% CI = 0.58~0.94) compared to the control group in females. There were also decreased percentages of the AG+GG genotype in the GD (*p* = 0.015 OR = 0.62, 95% CI = 0.43~0.91) and AITD (*p* = 0.027, OR = 0.67, 95% CI = 0.47~0.96) groups than in the control group in females (Table 2). There was no significant difference in allelic or genotype frequencies of rs2893321 between the GD, HT, or AITD and the control groups in males.

Meanwhile, there were no significant differences in genotype or allelic frequencies of rs1041569 between the GD and control groups, between the HT and control groups, or between the AITD and control groups. The allelic frequencies and genotypes of rs1041569 in females and males were also examined. There was no significant difference in allelic or genotype frequencies of rs1041569 between the GD and control groups, between the HT and control groups, or between the AITD and control groups in females or males (Table B in S1 File).

### Associations of rs1041569 and rs2893321with TSHRAb and AmiA titers

Correlations of thyroid function of the GD and HT groups at the baseline with the two SNPs were assessed. There was no significant association of the severity of GD and HT with the two SNPs in either the total population or in different genders (Table 3).

**Table 3:** Associations of thyroid function at the baseline with gene polymorphisms in Graves’ disease (GD) and Hashimoto’s thyroiditis (HT).

	HT		GD	
	Free T4 (ng/dl)	TSH (μU/ml)	Free T4 (ng/dl)	TSH (μU/ml)
rs1041569				
Females				
AT+TT	0.96±0.73	13.25±17.52	4.55±1.88	< 0.03
AA	0.81±0.29	18.17±19.47	4.25±1.68	< 0.03
*p* value	0.453	0.332	0.375	-
Male				
AT+TT	-	-	4.56±1.85	< 0.03
AA	0.90±0.71	18.2±19.5	4.00±1.96	< 0.03
*p* value	-	-	0.332	-
rs2893321				
Female				
AG+GG	0.93±0.71	12.50±17.57	4.29±1.84	< 0.03
AA	0.90±0.58	17.91±18.59	4.80±1.70	< 0.03
*p* value	0.822	0.711	0.079	-
Male				
AG+GG	0.42±0.53	216.52±244.65	4.58±1.72	< 0.03
AA	0.63±0.19	38.72±62.33	4.14±2.16	< 0.03
*p* value	0.498	0.834	0.344	-

Free T4 and thyroid-stimulating hormone (TSH) values are expressed as the mean±standard deviation.

**Fig 1:**
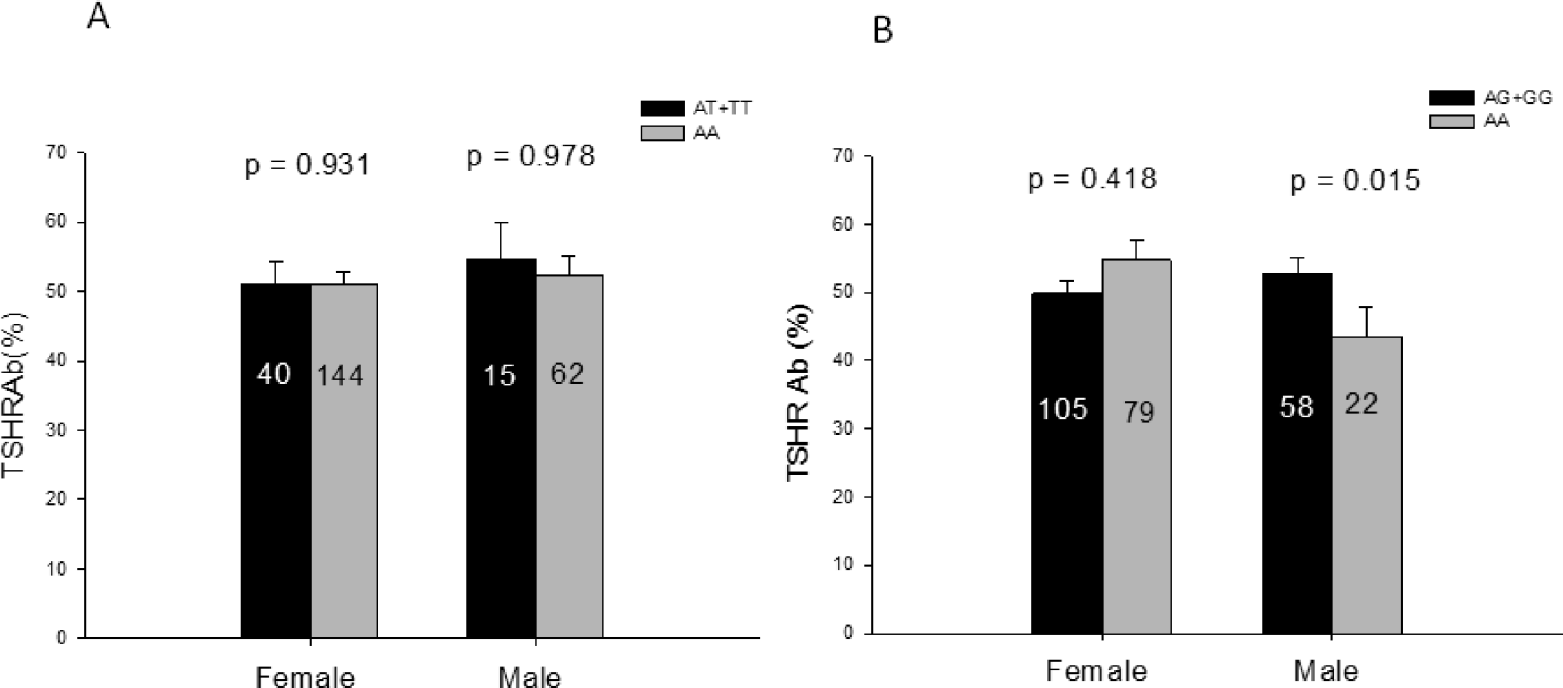
Thyroid-stimulating hormone (TSH) receptor antibody titers at the baseline in different genotypes of rs1041569 (Panel A) and rs2893321 (Panel B) in Graves’ disease in females and males. TSHRAb, thyroid-stimulating hormone receptor antibody; the number in the column indicates the number of patients.

There was no significant difference in TSHRAb titers at the baseline in patients with GD between the AT+TT and AA genotypes of rs1041569 or between the AG+GG and AA genotypes of rs2893321 (*p* = 0.964 and 0.590, respectively, data not shown). Association of TSHRAb levels with the two SNPs were determined in females and males. In males, the TSHRAb level was lower in the AA genotype than the AG+GG genotype of rs2893321 (*p* = 0.015), while there was no difference between the AA and AT+TT genotypes of rs1041569 (p = 0.978). No association of TSHRAb levels with the two SNPs was found in females (Fig 1).

**Fig 2:**
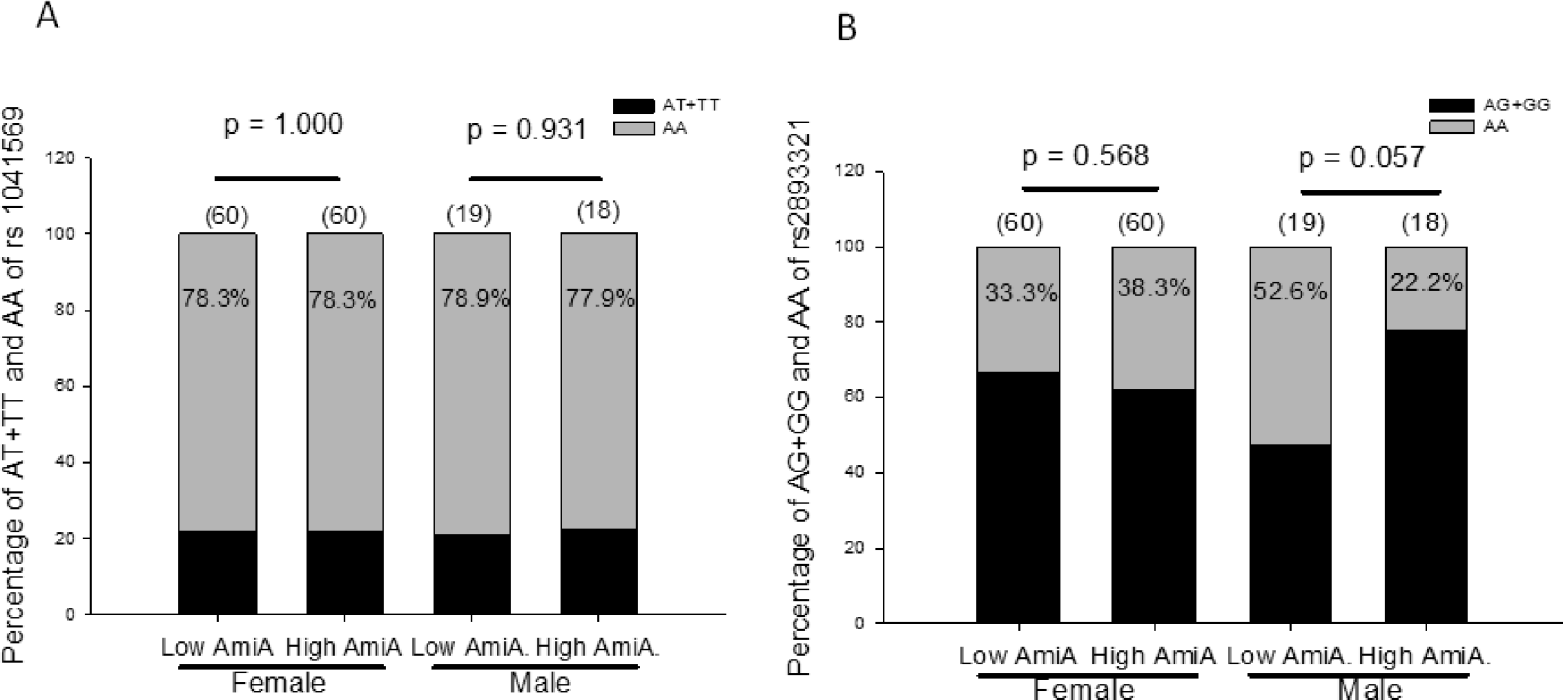
Anti-microsomal antibody (AmiA) titers at the baseline in different genotypes of rs1041569 (Panel A) and rs2893321 (Panel B) in autoimmune thyroid disease in females and males. Low AmiA, low AmiA titer; High AmiA, high AmiA titer; the number in the parenthesis indicates the number of patients; the number in the column indicates the frequency of the genotype.

There was no significant association of AmiA titers with rs1041569 or rs2893321 (*p* = 0.967 and 0.662, respectively, data not shown) in patients with AITD at the baseline. In males, there was a marginally higher percentage of the AA genotype of rs2893321 in the low-AmiA group than that in high-AmiA group (*p* = 0.057), but not of rs1041569 (*p* = 0.967). On the other hand, there was no association of rs1041569 or rs2893321 with AmiA titers in females (Fig 2).

### Prevalences of rs1041569 and rs2893321 with diversity of autoantibody production in AITD

**Fig 3:**
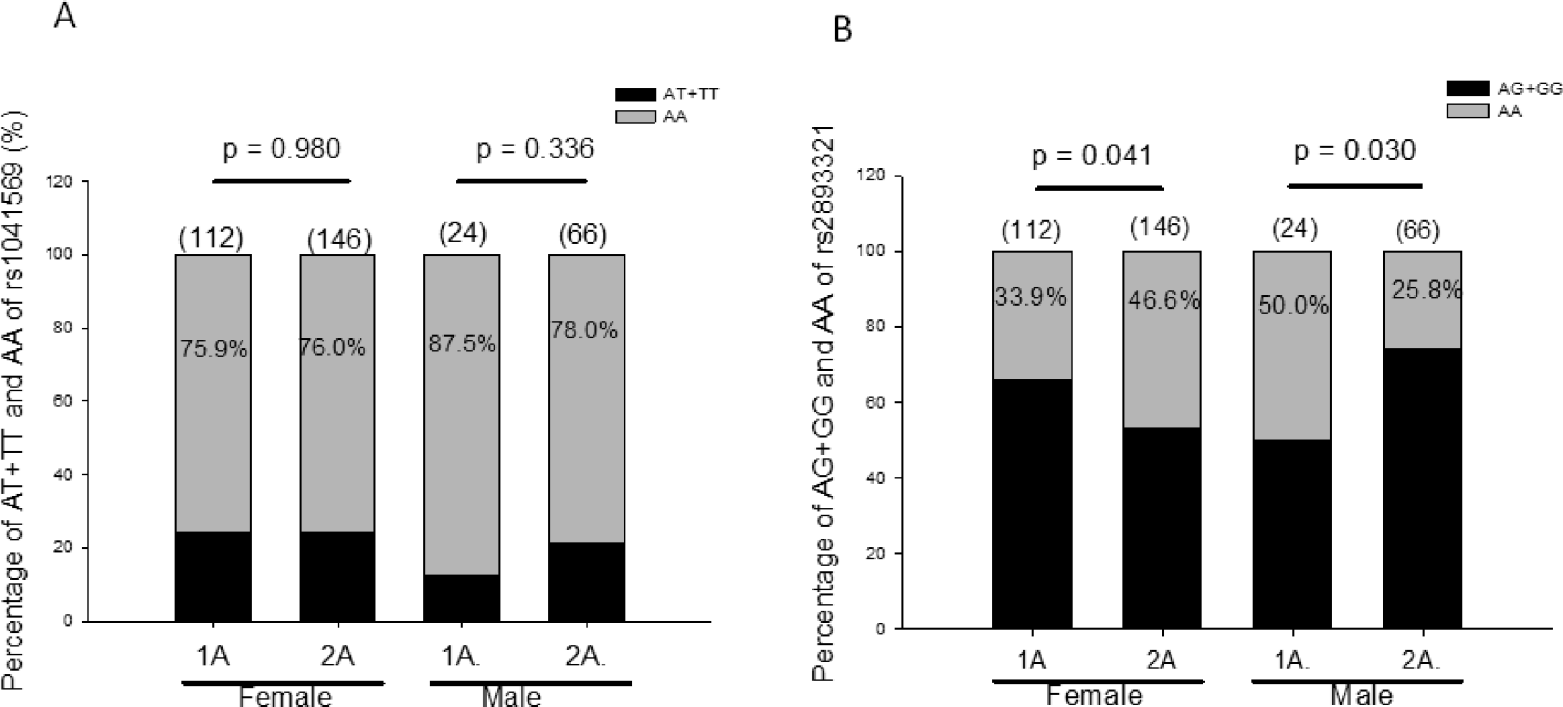
Prevalence of different genotypes of rs1041569 (Panel A) and rs2893321 (Panel B) in existing types of thyroid autoantibody in autoimmune thyroid disease in females and males. 1A, Grave’s disease but without the anti-thyroglobulin and anti-microsomal antibody or those with Hashimoto’s thyroiditis (one type of antibody); 2A, Graves’ disease with either the anti-thyroglobulin or antimicrosomal antibody or both (two types of antibody); the number in the parenthesis indicates the number of patients; the number in the column indicates the frequency of the genotype.

Increased BAFF levels allow autoreactive B cells to escape cell death, resulting in increased germinal center formation and autoantibody production. This suggests that the BAFF may also contribute to the diversity of autoantibody production. To test this hypothesis, we examined the association of these two SNPs of the BAFF with existing types of the TAb. Patients with AITD were stratified into two groups: one type of autoantibody (1A) and two types of autoantibody (2A) groups. The 1A group included patients with either TSHRAb or Hashimoto’s autoantibody (AmiA, ATA or both), while the 2A group included patients with both TSHRAb and Hashimoto’s autoantibody. There was no significant association of rs1041569 or rs2893321 with the existing types of TAb (*p* = 0.801 and 0.534, respectively). Furthermore, associations of the two SNPs with existing types of TAb in different genders were examined, and results are shown in Fig 3. In males, there was a higher frequency of the AA genotype of rs2893321 in group 1A than that in group 2A (*p* = 0.030). In the meanwhile, the AA genotype of rs2893321 was associated with the formation of 2A in females (*p* = 0.041). There was no significant association of the rs1041569 genotype with types of TAb in males or females.

### Associations of rs2893321 and rs1041569 with serum BAFF protein levels

Subjects in the AITD group had higher serum BAFF levels than those in the control group (*p* < 0.001. Figure C1 in S1 File). There was no difference in serum BAFF protein levels between the AA and AG+GG genotypes of rs2893321 in the AITD group (*p* = 0.356, Figure C2 in S1 File). There was also no significant difference in BAFF protein levels between the AA and AG+GG genotypes of rs2893321 in females or males (*p* = 0.145 and 0.465, respectively, data not shown). At the same time, there was no significant difference in serum BAFF protein levels between the AA and AT+TT genotypes of rs1041569 in AITD subjects (*p* = 0.917, data not shown). There was also no significant difference in serum BAFF protein levels between the AA and AT+TT genotypes of rs1041569 in females or males (*p* = 0.940 and 0.762, respectively, data not shown).

### Multivariate logistic regression analysis for susceptibility to GD, HT, and AITD

Demographic data, such as age, gender, FH, and smoking, were first included in the univariate analysis to predict the development of AITD, GD, or HT, and factors of interest together with rs2893321 were analyzed by a multivariate logistic regression. rs2893321 was still retained in the model for predicting AITD and GD (Table 4; *p* = 0.031, adjusted OR = 0.68, 95% CI = 0.48~0.97; *p* = 0.029, adjusted OR = 0.68, 95% CI = 0.48~0.96, respectively), but it was not selected for the model to predict the occurrence of HT (*p* = 0.192, adjusted OR = 0.68, 95% CI = 0.39~1.21). At the same time, age was also included in the univariate analysis to evaluate TSHRAb levels, AmiA titers, and 1A or 2A formation in each gender. As there was no association of age with AmiA or TSHRAb levels in either gender, age was not included in the multiple regression analysis to adjust the association of rs2893321 with the AmiA or TSHRAb level in either gender. On the contrary, age was selected as a significant risk factor in the univariate analysis to predict 1A and 2A in females, but not in males. After adjusting for age, rs2893321 was still retained in the model to predict 2A formation in females (*p* = 0.043, OR = 0.59, 95% CI = 0.35~0.98, Table C in S1 File).

**Table 4:** Multivariate logistic regression analysis to predict the development of Graves’ disease, Hashimoto’s thyroiditis, and autoimmune thyroid disease.

	Graves’ disease AOR (95% CI)	Hashimoto’s thyroiditis AOR (95% CI)	Autoimmune thyroid disease AOR (95% CI)
rs2893321			
AG+GG	0.68 (0.48~0.97)*	0.68 (0.39~1.21)	0.68 (0.50~0.97)*
AA	1	1	1
Age	-	1.05 (1.02~1.07)*	1.02 (1.01~1.03)*
Gender	-	0.24 (0.11~0.52)*	0.51 (0.35~20.75)*
Smoking	2.32 (1.45~3.70)*	-	2.27 (1.45~3.55)*
Family history of thyroid disease	6.06 (3.67~10.0)*	3.20 (1.47~7.00)*	5.52 (3.4~9.0)*

* *p* < 0.05. AOR, adjusted odds ratio; CI, confidence interval.

## Discussion

In this study, we first demonstrate that genetic variants of the BAFF are associated with GD and AITD, and genetic impacts show a dimorphic pattern. The SNP, rs2893321, may be a susceptible genetic variant for the development of GD and AITD, which was apparent in females but not in males. The AA genotype of rs2893321 was associated with low titers of the TSHRAb and AmiA in males but not in females. At the same time, the AA genotype of rs2893321 was associated with 2A in females, while it was associated with 1A formation in males.

Previous studies established that the BAFF is a powerful stimulator of B-lymphocyte biological functions, and dysregulation of the BAFF can lead to the development of AIDs [6,23]. Several studies were conducted to link genetic variants of the BAFF to AID development; however, no study has investigated the genetic association of AITD with the BAFF. In the current study, we demonstrated that frequencies of the G allele and AG+GG genotype of rs2893321 were lower in patients with GD and AITD than those in the control group, which suggests that rs2893321 is associated with a susceptibility to GD and AITD in this ethnic Chinese population. The lack of an association of the genetic variant of rs2893321 with HT might have been due to our limited population size. Additional studies with larger sample sizes are required to prove our findings. It is well known that the type 1 IFN signaling pathway is associated with immune function, and disruption or stimulation of the pathway can trigger several kinds of AIDs, including AITD [24,25,26,27,28]. Recently, we reported a genetic variant of interferon regulatory factor 8 in the development of HT and TAb formation [29]. In the present study, we further demonstrated that a genetic variant of the BAFF which was regulated by type 1 IFN can play a role in the occurrence and clinical phenotypes of AITD. Both studies implied a critical role of the type 1 IFN-BAFF signaling pathway in the development and clinical features of AITD, and therapy targeting the pathway may improve clinical management of AITD.

In this study, we also demonstrated that genetic impacts of the BAFF on the occurrence and phenotypes of both GD and AITD were gender-dependent. Briefly, the genetic effect of the rs2893321 variant on susceptibility to GD or AITD appeared to be weaker in males than in females, and the genetic actions of these variants might also be associated with suppression of TAb levels and inhibition of multiple kinds of autoantibody formation in males even if GD or AITD occurs. Gender-specific genetic effects in the susceptibility and phenotypes of type 1 diabetes (T1DM), GD, and multiple sclerosis were reported [30,31,32]. Recent advanced comprehensive research further demonstrated that associations of genetic variants with susceptibility to T1DM, RA, and myasthenia gravis are gender-deviation [33,34,35]. The molecular basis for these findings is unclear but might mainly be attributed to a discrepancy in basic immune responses between males and females, which results from distinct sex hormones [36]. Sex hormones are known to play direct roles in regulating immune functions and could function as modulators of the initiation, acceleration, and progression of AIDs, which results in the gender-deviation of AIDs [36]. In addition, close relationships between sex hormones and BAFF expression were reported in both human and animal studies. Pongratz et al. showed that a negative correlation existed between serum BAFF and testosterone levels in male patients with psoriatic arthritis, which implies an inhibitory effect of testosterone on BAFF function [37]. On the contrary, Panchanathan et al. recently demonstrated that administration of estrogen to cultured immunocytes from lupus-mice could increase BAFF gene expression, and administration of estrogen to a macrophage cell line induced BAFF gene expression, suggesting the regulatory role of estrogen in BAFF expression [38]. Moreover, Liu et al. also suggested that sex hormones might bind to loci near the SNP, followed by regulating some crucial gene expressions [34]. These findings support the notion that sex hormones might modify BAFF gene expression, or there is possible interplay between the BAFF and sex hormones at a certain level of the BAFF signaling pathway. However, further studies are needed to elucidate these issues.

A splicing variant of the BAFF, ΔBAFF, without a 57-bp coding region, was identified in humans (exon 3) and mice (exon 4) [39]. The ΔBAFF protein is known to play a role in suppressing BAFF's function, by reducing its biological function and release of the full-length BAFF protein, and by integrating with the BAFF protein to form heterotrimers, which limits the binding capacity of the BAFF to the BAFF receptor [39,40]. Roescher et al. first reported that treatment with ΔBAFF reduced the BAFF protein, diminished lymphocytes infiltrating in the salivary gland, and ameliorated the salivary amount in an SS-mouse model, and suggested that ΔBAFF might be a target for treating AIDs [40]. Interestingly, the SNP, rs2893321l, is located in an intron which is adjacent to exon 3. Simultaneously measuring serum BAFF and ΔBAFF levels would clarify the genetic role of rs2893321 in the BAFF and ΔBAFF. However, due to no available commercial kits being available to quantify ΔBAFF, only the serum BAFF protein was determined in this study. Unfortunately, we were unable to show the difference in serum BAFF protein levels between the AA and AG+GG genotypes of rs2893321. This observation might have resulted from some blood samples being obtained from patients in an inactive status, which would be expected to have reduced serum BAFF levels compared to those in an active stage.

In conclusion, to our best knowledge, this is the first study to demonstrate a possible association of genetic variants of the BAFF with the occurrences of GD and AITD, and differences in the concentration of the TAb and the kinds of TAb in males and females. However, we should point out certain limitations of the study. First, certain demographic data such as FH and smoking were not obtained from some patients. Further study with complete demographic data could make our results more convincing. Second, rs2893321 is located in the intron, which restricts the possibility of a functional assay. Finally, the aforementioned intronic SNP, rs2893321, is located close to exon 3, so it is still possible that other SNPs of the BAFF, especially near the coding region, such as exon 3, are associated with the formation of ΔBAFF and alterations of BAFF's function, and subsequently the development of GD or AITD [41]. Our future work is to investigate associations of other SNPs located in exons or the promoter region of the BAFF with GD or AITD and to measure serum BAFF and ΔBAFF levels to determine associations of the genetic variants and BAFF/ΔBAFF levels with GD or AITD.

## Supporting Information

S1 File Supplementary Figures and Tables. (PDF)

## Abbreviations

AITD: autoimmune thyroid disease
AmiA: anti-microsomal antibody
ATA: anti-thyroglobulin antibody
BAFF: B-lymphocyte-activating factor
GD: Graves’ disease
HT: Hashimoto’s thyroiditis
SNP: single-nucleotide polymorphism
SS: Sjögren's syndrome
TAb: thyroid autoantibody
TSHRAb: thyroid-stimulating hormone receptor antibody
