# Supplementary material for: Analysis of Associations of Human BAFF Gene Polymorphisms with Autoimmune Thyroid Diseases
Source: PLoS One. 2016 May 2;11(5):e0154436. doi: 10.1371/journal.pone.0154436 (PMC4852922; doi:10.1371/journal.pone.0154436)
Supplement: S1 File — (PDF) [file pone.0154436.s001.pdf]

S1 Fig A. Sequence of B-lymphocyte activating factor single-nucleotide polymorphism of rs2893321.

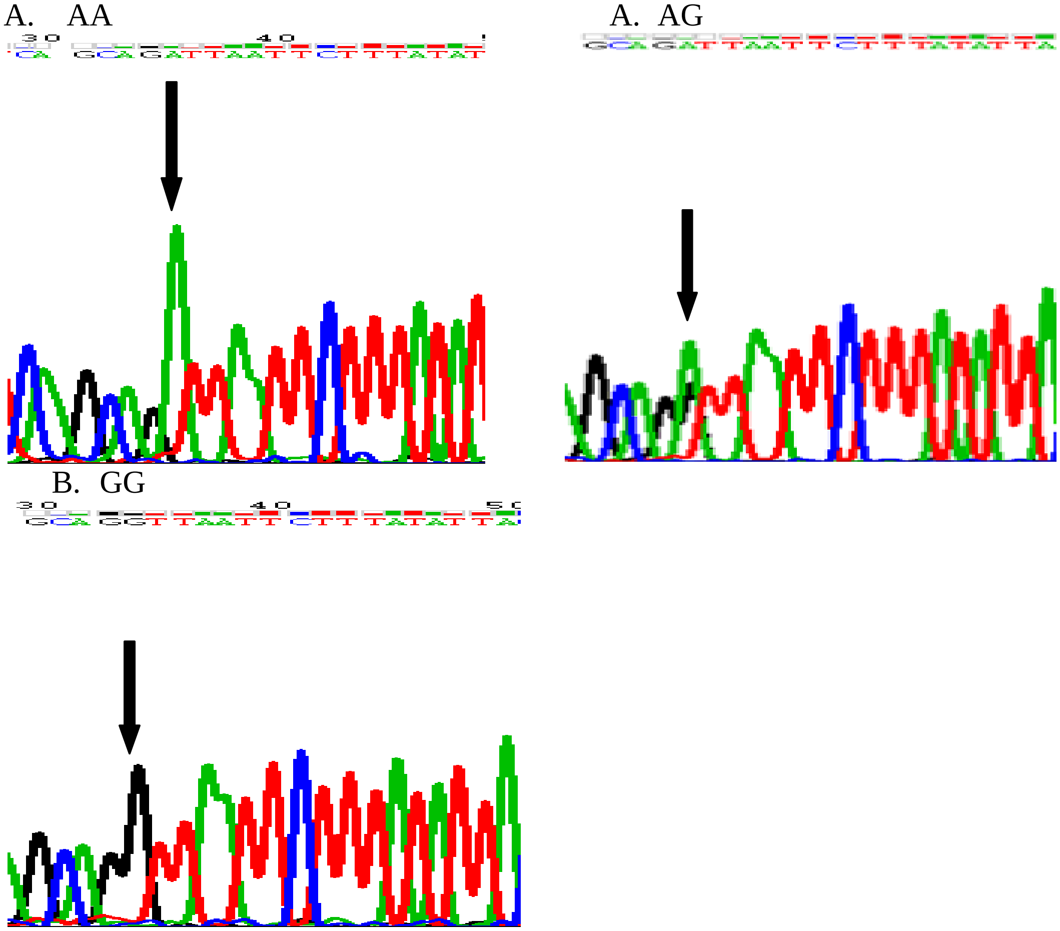

S1 Fig B. Sequences of B-lymphocyte activating factor single-nucleotide polymorphism (SNP) rs1041569.

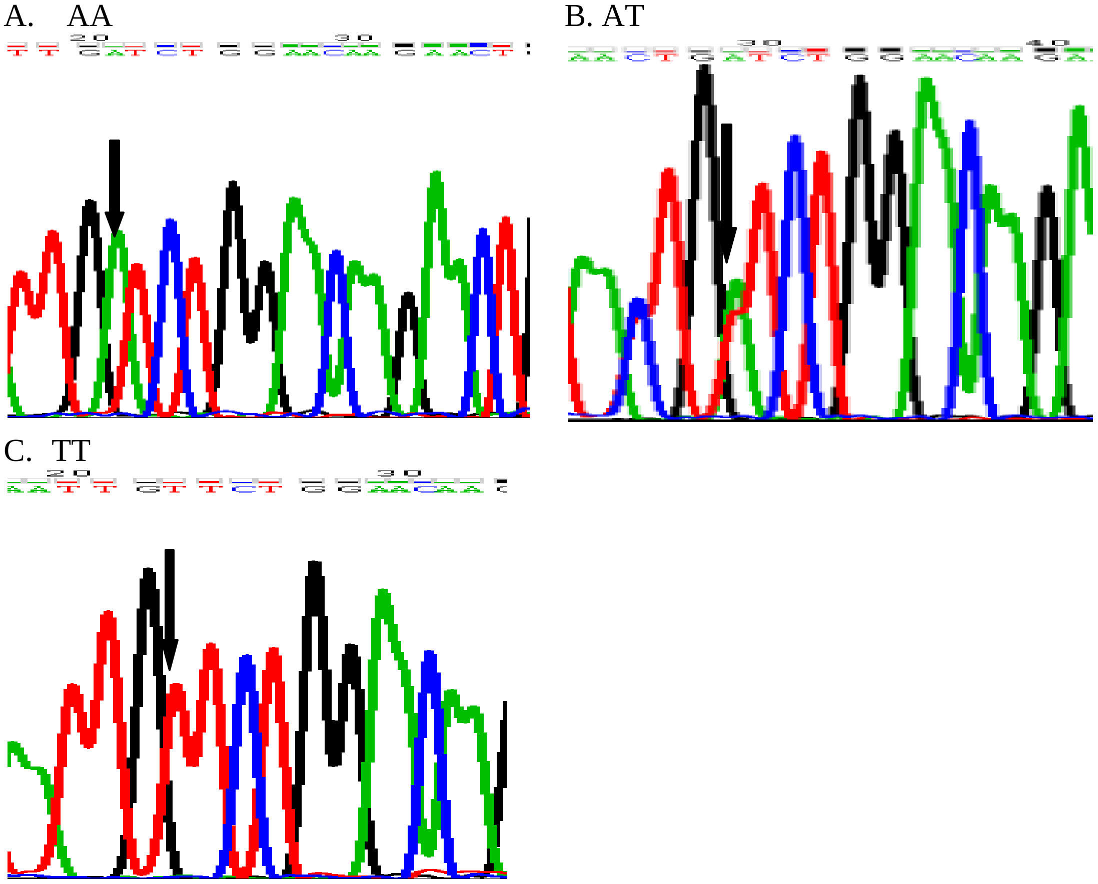

S1 Fig C. The comparison of serum BAFF levels between subjects in autoimmune thyroid disease (AITD) and those in the control group (Panel 1); The comparison of serum BAFF protein level between AA and AG+GG genotypes in AITD and control groups, respectively (Panel 2).

1.

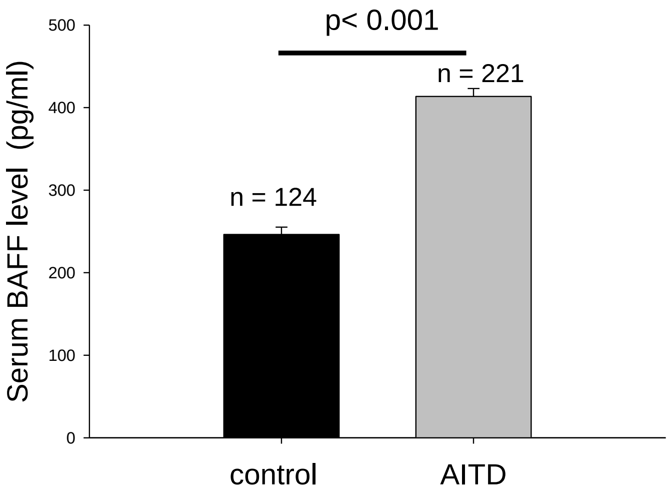

2

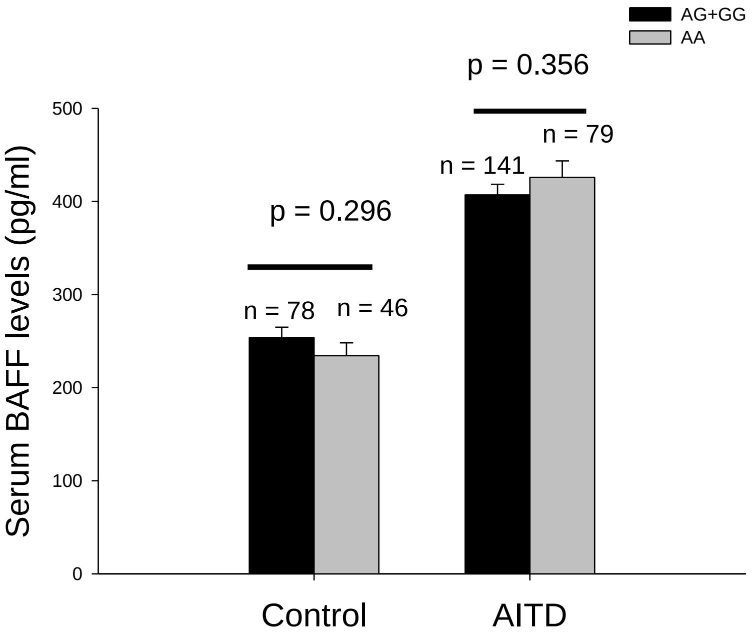

S1 Table A . Single-nucleotide polymorphisms (SNPs) in the B-lymphocyte activating factor (BAFF)

SNP	Locus	Location in the BAFF gene	Base change	Primer sequence	RFLP enzyme
rs2893321	108290686	Intron	A/G	F: TTTTTCGTTGGACTTGGTCA<br>R: CAACCCAAATCCAGAATCCT	Ase I
rs1041569	108267195	Promoter	A/T	F: CAATTCCCATACGAATTCCA<br>R: CCTCCATCAACCTTCTCAGG	DPNII

RFLP, restriction fragment length polymorphism.

S1 Table B. Genotype and allele frequencies of rs1041569 in the B-lymphocyte activating factor gene

Polymorphism	Control *n* (%)	GD *n* (%)	HT *n* (%)	AITD *n* (%)	OR1 (95% CI)	OR2 (95% CI)	OR3 (95% CI)
Total							
AA	268 (72.6)	244 (77.0)	65 (78.3)	309 (76.3)	1	1	1
AT	97 (26.3)	70 (22.1)	18 (21.7)	88 (22.5)	0.79 (0.56~1.13)	0.77 (0.43~1.36)	0.79 (0.57~1.10)
TT	4 (1.1)	3 (0.9)	0 (0.0)	3 (1.2)	0.82 (0.18~3.72)	-	0.65 (0.14~2.93)
AT+TT	101 (27.4)	73 (23.0)	18 (21.7)	79 (23.7)	0.79 (0.56~1.12)	0.73 (0.42~1.30)	0.78 (0.56~1.08)
Allele							
A	633 (85.8)	558 (88.0)	148 (89.2)	706 (88.3)	1	1	1
T	105 (14.2)	76 (12.0)	18 (10.8)	94 (11.7)	0.82 (0.60~1.12)	0.43 (0.43~1.25)	0.80 (0.60~1.08)
Female							
AA	177 (72.8)	170 (76.2)	57 (76.0)	227 (76.2)	1	1	1
AT	65 (26.7)	50 (22.4)	18 (24.0)	68 (22.8)	0.80 (0.52~1.22)	0.86 (0.47~1.57)	0.82 (0.55~1.21)
TT	1 (0.5)	3 (1.4)	0 (0.0)	3 (1.0)	3.12 (0.32~30.3)	-	2.34 (0.24~22.7)
AT+TT	66 (27.2)	73 (27.8)	18 (24.0)	71 (23.8)	0.84 (0.55~1.27)	0.85 (0.47~1.54)	0.84 (0.57~1.24)
Allele							
A	419 (86.2)	390 (87.4)	132 (88.0)	522 (87.6)	1	1	1
T	67 (13.8)	56 (12.6)	18 (12.0)	74 (12.4)	0.90 (0.61~1.31)	0.85 (0.49~1.49)	0.89 (0.62~1.26)
Male							
AA	91 (72.2)	74 (78.7)	8 (100)	82 (80.4)	1	1	1
AT	32 (25.4)	20 (21.3)	0 (0)	20 (19.6)	0.77 (0.41~1.45)	-	0.69 (0.39~1.38)
TT	3 (2.4)	0 (0.0)	0 (0.0)	0 (0.0)	-	-	-
AT+TT	35 (27.8)	20 (21.3)	0 (0)	102 (19.6)	0.70 (0.38~1.32)	-	0.67 (0.36~1.25)
Allele							
A	214 (84.9)	168 (89.3)	16 (100.0)	184 (90.2)	1	1	1

GD, Graves' disease; HT, Hashimoto's thyroiditis; AITD, autoimmune thyroid disease (Graves' disease + Hashimoto's thyroiditis); control, control group.
OR1, odds ratio 1, GD vs. the control.
OR2, odds ratio 2, HT vs. the control.
OR3, odds ratio 3, AITD vs. the control.
CI, confidence interval.
* $p < 0.05$.

S1 Table C. Multivariate logistic regression analysis to predict the development of the two existing types of thyroid autoantibodies in autoimmune thyroid disease in females and males

	Females<br>AOR (95% CI)	Males<br>AOR (95% CI)
rs2893321		
AG+GG	0.59 (0.35~0.98)*	2.88 (1.09~7.62) *
AA	1	1
Age	0.98 (0.96~1.00)*	-
Smoking	-	-
Family history of thyroid disease	-	-

* $p < 0.05$. AOR, adjusted odds ratio; CI, confidence interval.
